# Multiple Extramedullary-Bone Related and/or Extramedullary Extraosseous Are Independent Poor Prognostic Factors in Patients With Newly Diagnosed Multiple Myeloma

**DOI:** 10.3389/fonc.2021.668099

**Published:** 2021-07-07

**Authors:** JingSong He, XiaoYan Yue, DongHua He, Yi Zhao, Yang Yang, GaoFeng Zheng, Enfan Zhang, XiaoYan Han, WenJun Wu, Li Yang, Jing Chen, Zhen Cai

**Affiliations:** ^1^ Bone Marrow Transplantation Center, The First Affiliated Hospital, School of Medicine, Zhejiang University, Hangzhou, China; ^2^ Institute of Hematology, Zhejiang University, Hangzhou, China; ^3^ Zhejiang Laboratory for Systems & Precision Medicine, Zhejiang University Medical Center, Hangzhou, China

**Keywords:** extramedullary multiple myeloma, extramedullary-bone related, extramedullary extraosseous, PFS, OS

## Abstract

**Background:**

Extramedullary (EM) lesions are common in multiple myeloma (MM) and are often related to the poor prognosis of MM but are scarcely understood.

**Methods:**

In this retrospective study, the baseline characteristics of 357 newly diagnosed patients with extramedullary multiple myeloma (EMM) and their impact on the prognosis were analyzed. All patients received first-line treatment with bortezomib-based regimen.

**Results:**

The overall incidence rate of EM was 22.4%, and the detection rate of PET/CT was significantly higher than other imaging methods (P = 0.015). The cohorts consisted of 10 cases of extramedullary extraosseous (EME) and 70 cases of extramedullary-bone related (EMB), including 53 cases with single site involvement (one case with EME) and 27 cases with multiple sites (>1 site) involvement (nine cases with EME). EMM patients had high levels of hemoglobin (Hgb, ≥10 g/dl) and serum lactate dehydrogenase (LDH, >245u/L) and are inclined to early-stage revised international staging system (R-ISS). Compared to patients without EM, those with EMM had worse progression-free survival (PFS) (P = 0.014) and overall survival (OS) (P = 0.032). In addition, patients without EM and those with a single site of EMB had similar PFS and OS, while patients with multiple sites of EMB or EME and multiple sites of EMB with EME had poor PFS and OS. Multivariate analysis confirmed that multiple sites of EMB and/or EME were independent prognostic predictors affecting PFS and OS in newly diagnosed MM patients.

**Conclusions:**

This study suggested that among patients treated with bortezomib-based regimens, multiple sites of EMB and/or EME are independent poor prognostic factors for newly diagnosed MM patients, while a single site of EMB does not affect the survival of newly diagnosed MM patients. Thus, these findings could be used as a reference for the study of EMM patients in the new drug era, but prospective clinical studies are needed to provide evidence-based data for the diagnosis and treatment of EMM.

## Introduction

Multiple myeloma (MM) is a type of hematological malignant tumor characterized by abnormal proliferation of malignant plasma cells and secretion of a large number of monoclonal immunoglobulins, which cause a series of clinical symptoms, such as anemia hypercalcemia, and renal damage, and bone destruction (1). Most of the tumor cells are confined to the bone marrow, and in some cases, malignant plasma cells can break through the bone marrow and bone tissue involving periosteal tissue, forming tumorous masses in the adjacent bone site. The cells might also enter the blood circulation and colonize the distant tissue to form tumorous masses, known as extramedullary multiple myeloma (EMM). Among these, the adjacent bone is called extramedullary-bone related (EMB), while the extramedullary plasmacytoma distal from the bone is known as extramedullary extraosseous (EME) (2). Extramedullary (EM) lesions are also common in MM, but our understanding of the phenomenon is limited.

EMM appears in the diagnosis of MM or in the recurrence of the disease, and the incidence of EMM at diagnosis is about 3–30% in the diagnosis, while it increased to 6–40% in the refractory recurrence of the disease. The incidence of EMB is 6–35%, and the incidence of EME is 0.5–3.5%. Currently, there is not a precise definition of EMD and a lot of controversies (2, 3); also, periods, inspection methods, and the detection rates of each report were different (2, 4–7). In addition, there were limited data on the baseline characteristics of EMM, such as prevalence, clinical and laboratory characteristics, and the efficacy of new drugs. The present study aimed to analyze the incidence, clinical characteristics, and survival status of patients with EMM treated with a bortezomib-based regimen at the time of diagnosis in the real-world data of 357 patients with newly diagnosed MM. Also, PET/CT findings served as a reference to study the patients with EMM in the era of new drugs.

There are two cases: one is solitary plasmacytoma (SP) in which a single EM lesion is present in patients with no bone marrow plasma cell involvement or only a minimal bone marrow involvement (<10%), and the difference between SP and EMM is that SP patients cannot be diagnosed as MM and cannot be attributed to EMM. Another case is that of malignant plasma cells involving peripheral blood up to 2,000 cells/ml or account for ≥20% of peripheral blood nucleated cells (2), known as plasma cell leukemia, which is an extreme state of EME with poor survival. Patients with the above two conditions were excluded from this study, although plasma cell leukemia should also belong to EMM.

## Methods

### Patients and Treatment

This study was a retrospective analysis and was approved by the Ethics Committee of the First Affiliated Hospital of the Medical School of Zhejiang University [ethics number of (2021) IIT (078)]. From May 2013 to June 2019, patients who were newly diagnosed according to the World Health Organization (WHO) or the International Myeloma Working Group (IMWG) diagnostic criteria in our center were included if they had symptomatic or active MM with detectable M protein in the blood and/or urine and received first-line treatment as bortezomib-based regimen. At least one course of treatment was completed, and the efficacy was evaluated. From the beginning of the treatment to the end of follow-up, all patients were informed of their condition and survival status through inpatient services, outpatient services, or telephone contact.

All patients received a bortezomib-based regimen either as a two-drug combined PD regimen (bortezomib combined with dexamethasone) or a three-drug combined regimen consisting of PD plus a third drug that was cyclophosphamide (PCD), adriamycin (PAD), thalidomide (PTD), or lenalidomide (PRD). The specific administration method is detailed elsewhere (8). According to the patient’s age, physical condition, and willingness, partial remission (PR) was obtained after at least three to four courses of induction treatment; then, autologous hematopoietic stem cell transplantation (ASCT) was performed. After induction therapy with or without ASCT, patients received maintenance therapy of bortezomib, lenalidomide, or thalidomide.

### Response

The IMWG efficacy evaluation standard was used to assess the efficacy of the treatment, including complete remission (CR), very good partial remission (VGPR), PR, stable disease (SD), and progressive disease (PD) (9, 10). Progression-free survival (PFS) was defined as the time from the beginning of the patients’ first course of treatment to disease progression, death, or the final follow-up visit, while the overall survival (OS) was calculated from the time of the first course of treatment to death or the final follow-up visit.

### Data Acquisition

All patients were hospitalized during the initial diagnosis and treatment. The majority of the patients underwent PET/CT examination before starting the initial treatment. The remaining patients only underwent local MR (including thoracolumbar vertebrae and/or skull or symptomatic site) or CT examination (chest or abdomen) and B ultrasound (hepatobiliary, spleen and pancreas, retroperitoneal, bilateral ureter, and bladder, including prostate in men and uterus and accessories in women) due to economic reasons. Data regarding the above imaging, Durian-Salmon staging, international staging system (ISS), bone marrow examination results, and hematuria test results were obtained from the hospital information system. A retrospective evaluation of the patient was performed based on the patient’s ISS stage, serum lactate dehydrogenase level (LDH), and the obtained fluorescence *in situ* hybridization (FISH) results, in which 1q amplification was also regarded as a genetic abnormality of poor prognosis (11).

According to the wishes of patients and their families, the bone marrow cells of most patients were detected by FISH for specific chromosomal abnormalities. Specifically, 8–10 ml of the patient’s heparin anticoagulated bone marrow was obtained by bone marrow puncture, mixed with an equal volume of lymphocyte separation solution, and bone marrow mononuclear cells obtained by density gradient centrifugation. Subsequently, CD138-positive myeloma cells were obtained by CD138 immunomagnetic bead-positive sorting. FISH was used to examine the bone marrow cells for specific chromosomal abnormalities, including del-(17p13), 1q21 gain, del(13q14), and 14q32 rearrangement, and specific translocations, including t (4,14), t(11;14), and t(14;16) in a small number of patients with 14q32 rearrangement. After hybridization with relevant probes, 400 interphase cells were counted under a fluorescence microscope. The number of cells with ≥3 copies (known as 1q gain/amplification) of the signal at 1q, the number of cells with 17p and 13q <2 copies, and the number of cells with 14q signal separation were counted, and the corresponding proportion of positive cells was calculated. As a result, the positive threshold of 14q32 rearrangement was 10%, and the positive threshold for 1q gain/amplification, del(13q), and del(17p) was set to 20%.

### Statistical Analysis

All patients were followed up until June 30, 2020, and the efficacy of treatment was evaluated after completing each course. The threshold of acquiring data was based on the literature: the normal threshold value of our center or the receiver operating characteristic (ROC) curve. Mann–Whitney test was used to compare the clinical features between groups, and the chi-square test (Fisher’s exact test) was employed for comparison of classification data. The Kaplan–Meier method was used to generate survival curves, and the log-rank test was used to compare the differences in patient survival. Univariate analysis of age, Durie-Salmon stage, ISS stage, R-ISS stage, bone marrow plasma cell ratio, blood creatinine (Cr) level, blood LDH level, and different EMM situations examined the impact of these variables on patient survival. All the test results were bilateral. P-value <0.05 indicated statistical significance, and factors with P-values <0.1 were assessed by multivariate analysis. The results of multivariate analysis using the Cox proportional hazards model are shown as the hazard ratios (HRs) and 95% confidence intervals (CIs). The statistical analyses were performed using SPSS for Windows 26.0.

## Results

### Clinical Characteristics of Patients With EM Involvement

**Figure 1:**
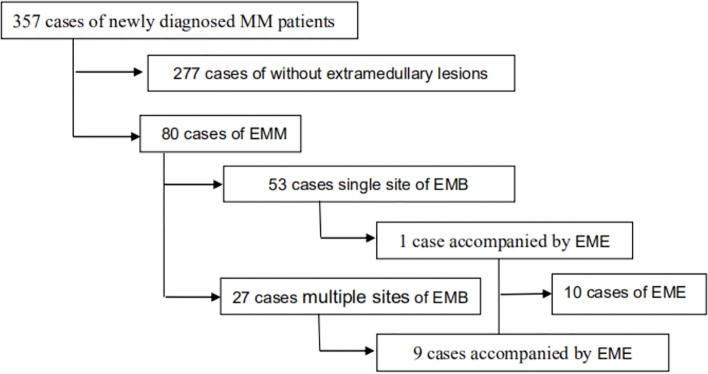
Schematic diagram of EMM patient detection.

A total of 357 newly diagnosed MM patients were enrolled in this study. The cohort consisted of 198 males and 159 females; the median age was 63 (range, 31–84) years. Among them, 80 (22.4%) patients showed EM lesions at the initial diagnosis. Moreover, 251 patients underwent systemic PET/CT examination at the time of diagnosis, suggesting that 65 (25.9%) cases had EM lesions, including 58 (23.1%) cases of EMB and 7 (2.8%) cases of EME. Among 106 patients, 15 (14.2%) cases showed EM lesions in other images or physical examinations, including 12 (11.3%) cases with EMB and 3 (2.8%) cases with EME (**Figure 1**), suggesting that PET/CT examination had a higher detection rate of EMM than other imaging methods (P = 0.015), but there was no significant difference in the detection of EME. EMB involved 48 cases of sternum and ribs, 19 cases of vertebral body, 14 cases of pelvis, 5 cases of skull, and 3 cases of limbs, while EME involved 5 cases of pleural effusion, 1 case of lung, abdominal organs (including space occupation in liver and spleen, space occupation in gastrointestinal), and 8 cases of lymph nodes.

**Table 1** lists the baseline characteristics and treatment specifics of all patients. The results showed that the hemoglobin (Hgb) level in patients with EMM was significantly higher than that in patients without EM, and a large number of patients with EMM had Hb ≥100 g/L (55.7 *vs.* 36.5%, P = 0.002) and were more inclined to the early stage of R-ISS (P = 0.009). In addition, more EMM patients had higher serum LDH levels than normal (32.5 *vs*. 16.2%, P = 0.001). However, no significant difference was detected in age, gender, ISS staging, type of M protein, peripheral blood platelet count, and cytogenetic abnormalities between EMM and non-EM groups.

**Table 1:** Clinical Characteristics of EMM and no EM patients at diagnosis (N = 357).

	MM without EMMn = 277	MM with EMMn = 80	P value
Age, n (%)			
Median (IQR)	63 (55–69)	61 (54–67.8)	0.260
≤68 years	206 (74.4)	61 (76.3)	0.733
>68 years	71 (25.6)	19 (23.8)	
Gender, n (%)			0.502
Male	151 (54.5)	47 (58.8)	
Female	126 (45.5)	33 (41.2)	
Type of M protein, n (%)			0.540
IgA	65 (23.5)	18 (22.5)	
IgG	131 (47.3)	34 (42.5)	
IgD	14 (5.1)	7 (8.8)	
Light chain	65 (23.5)	19 (23.8)	
Biphenotypic	2 (0.7)	2 (2.5)	
LDH			
Median (IQR)	188 (151–230)	200.5 (145.3–272.3)	0.125
Normal	232 (83.8)	54 (67.5)	0.001
Elevated	45 (16.2)	26 (32.5)	
D-S, n (%)			0.094
1 + 2	57 (20.6)	9 (11.3)	
3A	170 (61.4)	59 (73.8)	
3B	50 (18.1)	12 (15.0)	
ISS, n (%)			0.132
1	77 (28.7)	31(38.8)	
2	90 (32.5)	25 (31.3)	
3	110 (39.7)	24 (30.0)	
R-ISS, n (%)			0.009
1	19 (7.9)	15 (20.5)	
2	148 (61.4)	40 (54.8)	
3	74 (31.7)	18 (24.7)	
Unknow	86	39	
FISH			
1q gain/amp	108 (51.7)	27 (41.5)	0.159
Del 17p	13 (6.2)	2 (3.1)	0.533
Del 13q	73 (34.9)	15 (23.1)	0.094
14q rearrangement	74 (35.4)	18 (27.7)	0.294
Unknow	68	15	
Bone marrow plasma cell percentage			
Median (IQR)	28.5 (15.3–45.8)	22 (9.8–35.8)	0.023
≤30%	147 (53.1)	51 (63.8)	0.090
>30%	130 (46.9)	29 (36.2)	
Spleen			0.515
Normal	214 (77.3)	59 (73.8)	
Enlarged	63 (22.7)	21 (26.3)	
Hb(g/L)			
Median (IQR)	92 (74.5–109)	101 (85–124)	0.002
≥10	101 (36.5)	44 (55.7)	0.002
<10	176 (63.5)	35 (44.3)	
Plt(× 10^9^/L)			
Median (IQR)	172 (118–222)	169 (125.5–214.5)	0.866
≥150	168 (62.5)	44 (57.1)	0.399
<150	101 (37.5)	33 (42.9)	
CRP(g/L)			
Median (IQR)	2.1 (0.6–7.5)	2.7 (1.1–10.4)	0.115
≤8	193 (76.0)	52 (69.3)	0.247
>8	61(24.0)	23 (30.7)	
Therapy received			<0.001
PD	48 (17.3)	9 (11.3)	
PAD	18 (6.5)	28 (35.0)	
PCD	186 (67.1)	37 (46.3)	
PTD or PRD	25 (9.0)	6 (7.5)	
ASCT			0.404
No	242 (87.4)	67 (83.8)	
Yes	35 (12.6)	13 (16.3)	

MM, multiple myeloma; EMM, extramedullary multiple myeloma; IQR, interquartile range; LDH, lactate dehydrogenase; D-S, Durie-Salmon Staging; ISS, International Staging System; R-ISS, Revised International Staging System; FISH, fluorescence in situ hybridization; Hgb, hemoglobin; Plt, platelet; CRP, C-reaction protein; PD, bortezomib, dexamethasone; PCD, bortezomib, dexamethasone, cyclophosphamide; PAD, bortezomib, dexamethasone, adriamycin; PTD, bortezomib, dexamethasone, thalidomide; PRD, bortezomib, dexamethasone, lenalidomide; ASCT, Autologous hematopoietic stem cell transplantation.

Notably, 84 (23.5%) patients had no splenic space-occupying lesions but had spleen enlargement. Among them, 23 (21.7%) patients had spleen enlargement detected by B-ultrasound or CT, and 61 patients (24.3%) had spleen enlargement detected by PET/CT, of which 25 (10.0%) cases did not exhibit any increase in the standardized uptake value (SUV), 36 (14.3%) cases had an increase in SUV and no difference as detected in the incidence of spleen enlargement between the EMM and non-EM groups (P = 0.515).

### Effects of EM Involvement on Patient Survival

The median duration of follow-up was 36.1 months, the median PFS time was 34.4 (95% CI: 25.5–43.3) months, and the estimated 3-year and 5-year PFS rates were 49.2 and 31.8%, respectively. The median OS was 60.5 (95% CI: 54.1–66.9) months, and the estimated 3-year and 5-year OS rates were 70.7 and 50.5%, respectively. Compared to patients without EM, patients with EMM had worse PFS and OS (P = 0.014, 0.032). The median PFS and OS of patients with EMM were 21.8 (95% CI: 13.4–30.2) months and 44.0 (95% CI: 23.7–64.3) months, respectively; while the median PFS and OS of patients without EM were 38.1 (95% CI: 30.6–45.6) months and 61.4 (95% CI: 55.9–67.0) months, respectively.

Univariate analysis showed that the EME or EMM of >1 site extramedullary lesion indicated poor PFS (P < 0.001), and the 3-year PFS rates were 11.3 and 28.1%, respectively. However, no difference was detected in PFS between patients with single-site EMM and MM without EM (P = 0.662), with a 3-year PFS rate of 48.6 and 51.5%, respectively. The patient’s age, Durie-Salmon stage, ISS stage, R-ISS stage, peripheral blood platelet count, bone marrow plasma cell percentage, serum LDH level, types of M protein, and enlarged spleen significantly affected the PFS of the patients (P < 0.05) (**Tables 2** and **3**).

**Table 2:** Survival of MM patients.

Median Survival, m (95% CI)		PFS	P value	OS	P value
Gender	Male	29.6 (19.5–39.7)	0.116	/	/
	Female	42.9 (32.7–53.2)		/	/
Age	≤68 years	41.8 (34.6–49.0)	0.005	63.1(52.9–73.4)	0.005
	>68 years	21.2 (15.7–26.7)		44.0 (35.0–52.9)	
Type of M protein	non-IgD	38.1 (29.8–46.4)	<0.001	63.0 (57.3–68.6)	0.014
	IgD	16.3 (12.3–20.3)		38.2 (19.0–57.4)	
D-S	1 + 2+3A	38.7 (31.7–45.7)	0.019	63.1(52.7–73.5)	<0.001
	3B	18.6 (14.2–23.0)		33.4 (19.7–47.0)	
ISS	1 + 2	41.8 (34.1–49.5)	0.048	64.0 (52.2–75.8)	<0.001
	3	21.8 (11.7–31.9)		42.6 (34.2–50.9)	
RISS	1 + 2	38.1 (30.4–45.8)	0.002	64.8	<0.001
	3	18.9 (16.3–21.5)		36.2 (28.6–43.7)	
Hgb	≥100 g/L	38.7 (30.6–46.8)	0.203	64.8	0.018
	<100 g/L	28.4 (18.1–38.8)		56.8 (43.8–69.7)	
Plt	≥150 × 10^9^/L	42.8 (32.2–53.4)	0.001	63.0	0.001
	<150 × 10^9^/L	24.1 (15.4–32.8)		49.0 (40.1–57.9)	
Bone marrow plasma cells percentage	≤30%	45.4 (34.9–55.8)	<0.001	NR	<0.001
	> 30%	23.6 (17.5–29.7)		51.1 (39.0–63.3)	
Cr	≤177 umol/L	38.1 (30.6–45.6)	0.077	63.1 (52.8–73.5)	<0.001
	>177 umol/L	20.7 (16.7–24.6)		38.2 (23.4–52.9)	
LDH	≤245 u/L	42.8 (35.1–50.5)	<0.001	64.0 (52.3–75.6)	<0.001
	>245 u/L	18.8 (14.0–23.7)		32.0 (21.3–42.8)	
CRP	≤8 g/L	34.6 (24.6–44.5)	0.122	63.0 (53.3–72.6)	0.020
	>8 g/L	23.5 (16.3–30.7)		44.0 (33.6–54.3)	
Spleen	Normal	43.1 (34.6–51.5)	<0.001	64.8	<0.001
	Enlarged	18.6 (15.1–22.1)		32.0 (29.8–34.3)	
EMM			<0.001		<0.001
	Without	38.1 (30.6–45.6)		61.4 (55.9–67.0)	
	Single EMM	34.6 (5.2–63.9)		64.8 (23.9–105.7)	
	>1 EMM	10.4 (6.1–14.6)		25.0 (8.9–41.1)	
EMM			<0.001		<0.001
	Without	38.1 (30.6–45.6)		61.4 (55.9–67.0)	
	EMB	23.5 (2.8–44.2)		64.8 (34.3–95.3)	
	EME	5.9 (2.6–9.1)		14.9 (4.6–25.2)	
EMM			<0.001		<0.001
	Without	38.1 (30.6–45.6)		61.4 (55.9–67.0)	
	Single EMB	34.6 (5.4–63.7)		64.8 (24.0–105.6)	
	>1 EME or EMB	11.2 (7.6–14.8)		30.8 (21.6–40.0)	
	>1 EMB with EME	8.3 (3.6–13.0)		17.6 (9.6–25.7)	

CI, confidence interval; PFS, Progression-free survival; OS, Overall survival; D-S, Durie-Salmon Staging; ISS, International Staging System; R-ISS, Revised International Staging System; Hgb, hemoglobin; Plt, platelet; Cr, creatinine; LDH, lactate dehydrogenase; CRP, C-reaction protein; EMM, extramedullary multiple myeloma; EMB, extramedullary-bone related; EME, extramedullary extraosseous.

**Table 3:** PFS of MM patients.

Univariable analysis			Multivariable analysis		
Variable	HR (95% CI)	P value	Variable	HR (95% CI)	P value
Gender Female	0.79 (0.59–1.06)	0.116	Age > 68years	1.59 (1.13–2.25)	0.008
Age > 68 years	1.57 (1.14–2.16)	0.005	Type of M protein IgD	2.69 (1.52–4.77)	0.001
Type of M protein IgD	2.67 (1.66–4.30)	<0.001	RISS 3 *vs* 1 + 2	1.53 (1.08–2.16)	0.017
D-S 3B *vs* 1 + 2+3A	1.54 (1.07–2.22)	0.019	Bone marrow plasma cells percentage >30%	1.73 (1.25–2.41)	0.001
ISS 3 *vs* 1 + 2	1.35 (1.00–1.82)	0.048	Spleen enlarged	1.67 (1.18–2.36)	0.004
RISS 3 *vs* 1 + 2	1.68 (1.20–2.34)	0.002	EMM		
Hgb <100 g/L	1.21 (0.90–1.64)	0.203	Single EMB *vs* non-EM	1.24 (0.80–1.92)	0.338
Plt <150 × 10^9^/L	1.61 (1.20–2.16)	0.001	>1 EME or EMB *vs* non-EM	2.73 (1.46–5.13)	0.002
Bone marrow plasma cells percentage >30%	1.77 (1.32–2.37)	<0.001	>1 EME and EMB *vs* non-EM	12.48 (5.15–30.23)	<0.001
Cr >177umol/L	1.38 (0.96–1.99)	0.077			
LDH >245 u/L	2.08 (1.49–2.92)	<0.001			
CRP >8 g/L	1.30 (0.93–1.81)	0.122			
Spleen enlarged	2.15 (1.56–2.96)	<0.001			
EMM					
Single EMM *vs* non-EM	1.13 (0.76–1.70)	0.545			
>1 EMM *vs* non-EM	2.99 (1.85–4.85)	<0.001			
EMM					
EMB *vs* non-EM	1.30 (0.91–1.85)	0.152			
EME *vs* non-EM	8.54 (4.12–17.73)	<0.001			
EMM					
Single EMB *vs* non-EM	1.10 (0.72–1.66)	0.668			
>1 EME or EMB *vs* non-EM	2.40 (1.36–4.25)	0.003			
>1 EME and EMB *vs* non-EM	7.68 (3.54–16.66)	<0.001			

PFS, Progression-free survival; HR, hazard ratio; CI, confidence interval; D-S, Durie-Salmon Staging; ISS, International Staging System; R-ISS, Revised International Staging System; Hgb, hemoglobin; Plt, platelet; Cr, creatinine; LDH, lactate dehydrogenase; CRP, C-reaction protein; EMM, extramedullary multiple myeloma; EM, Extramedullary lesions; EMB, extramedullary-bone related; EME, extramedullary extraosseous.

During the follow-up, 128 (35.9%) patients were deceased, of which 33 (41.3%) died of EMM. Univariate analysis showed that EME or EMM of >1 site extramedullary lesion indicated poor OS (P < 0.001), and the 5-year OS rates were 0 and 29.5%, respectively. However, no difference was detected in OS between the single site of EMM and MM patients without EM (P = 0.790), and the 5-year OS rates were 59.2 and 51.7%, respectively. The patient’s age, Durie-Salmon stage, ISS stage, R-ISS stage, hemoglobin level, peripheral blood platelet count, bone marrow plasma cell percentage, serum creatinine, serum LDH level, serum C-reaction protein (CRP) level, types of M protein, and enlarged spleen significantly affected patient OS (P < 0.05) (**Tables 2** and **4**).

**Table 4:** OS of MM patients.

Univariable analysis			Multivariable analysis		
Variable	HR (95% CI)	P value	Variable	HR (95% CI)	P value
Gender Female	0.84 (0.59–1.20)	0.343	Age >68 years	1.64 (1.07–2.49)	0.022
Age >68 years	1.88 (1.29–2.72)	0.001	D-S 3B *vs* 1 + 2+3A	1.93 (1.13–3.28)	0.016
Type of M protein IgD	2.09 (1.15–3.80)	0.014	RISS 3 *vs* 1 + 2	1.76 (1.11–2.79)	0.017
D-S 3B *vs* 1 + 2+3A	2.61 (1.76–3.87)	<0.001	Bone marrow plasma cell percentage>30%	1.68 (1.11–2.55)	0.015
ISS 3 *vs* 1 + 2	2.19 (1.54–3.10)	<0.001	Spleen enlarged	1.91 (1.26–2.91)	0.003
RISS 3 *vs* 1 + 2	2.74 (1.88–4.01)	<0.001	EMM		
Hgb <100 g/L	1.55 (1.07–2.26)	0.020	Single EMB *vs* non-EM	1.35 (0.75–2.43)	0.316
Plt <150 × 10^9^/L	1.79 (1.27–2.54)	0.001	>1 EME or EMB *vs* non-EM	3.16 (1.55–6.44)	0.002
Bone marrow plasma cell percentage >30%	2.05 (1.45–2.92)	<0.001	>1 EME and EMB *vs* non-EM	6.88 (2.96–16.02)	<0.001
Cr >177 umol/L	2.45 (1.66–3.62)	<0.001			
LDH >245 u/L	2.54 (1.75–3.69)	<0.001			
CRP >8 g/L	1.56 (1.07–2.28)	0.020			
Spleen enlarged	2.63 (1.83–3.79)	<0.001			
EMM					
Single EMM *vs* non-EM	0.98 (0.58–1.67)	0.940			
>1 EMM *vs* non-EM	3.43 (2.03–5.81)	<0.001			
EMM					
EMB *vs* non-EM	1.19 (0.76–1.86)	0.450			
EME *vs* non-EM	8.74 (4.30–17.78)	<0.001			
EMM					
Single EMB *vs* non-EM	0.92 (0.54–1.59)	0.775			
>1 EME or EMB *vs* non-EM	2.52 (1.31–4.86)	0.006			
>1 EME or EMB *vs* non-EM	8.21 (3.90–17.32)	<0.001			

OS, Overall survival; HR, hazard ratio; CI, confidence interval; D-S, Durie Salmon Staging; ISS, International Staging System; R-ISS, Revised International Staging System; Hgb, hemoglobin; Plt, platelet; Cr, creatinine; LDH, lactate dehydrogenase; CRP, C-reaction protein; EMM, extramedullary multiple myeloma; EM, Extramedullary lesions; EMB, extramedullary-bone related; EME, extramedullary extraosseous.

**Figure 2:**
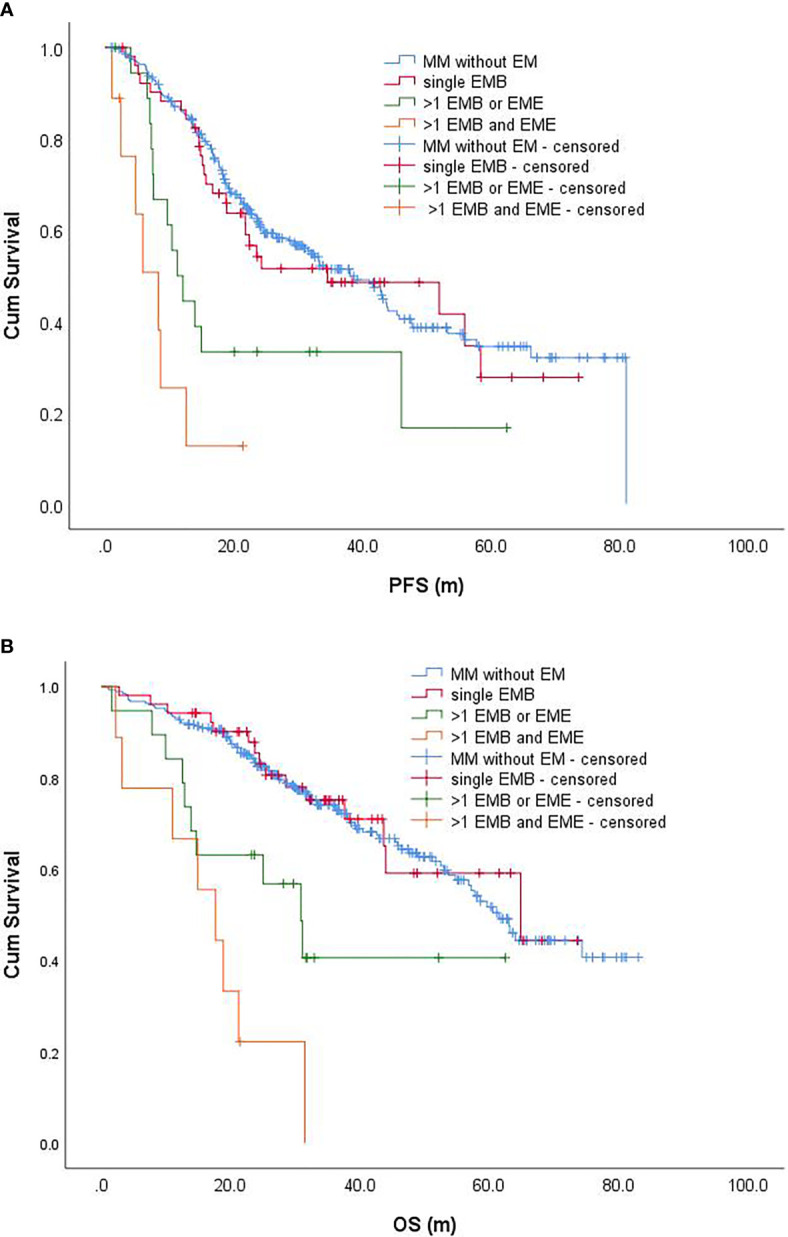
Progression-free survival **(A)** and overall survival **(B)** of patients with different EMMs and patients without EM. PFS, progression-free survival; OS, overall survival.

All patients with EME were accompanied by EMB. Among them, only one case was accompanied by a single site of EMB, and the remaining EME was accompanied by >1 site of EMB. The EMM was divided into a single site of the EMB group, >1 site of EMB or EME group, and >1 site of EMB with EME groups, with 52, 19, and 9 cases, respectively. Univariate analysis indicated that the above EM status significantly affected the PFS and OS of patients (P < 0.001, **Figures 2A, B**). Furthermore, patients with >1 site of EMB or EME, and patients with >1 site of EMB with EME (3-year PFS rates was 33.3 and 12.7%; 5-year OS rates was 40.6% and 0, respectively) had significantly worse PFS and OS compared to those without EM and patients with single site of EMB (3-year PFS rates were 51.1 and 48.6%; 5-year OS rates were 51.7 and 59.2%, respectively) (P < 0.001) (**Tables 2−** **4**).

Cox proportional hazards model was used for multivariate analysis. The factors that enter the analysis include age, Durie-Salmon stage, R-ISS stage, bone marrow plasma cell percentage, types of M protein, enlarged spleen, and the occurrence of EM. The results suggested that age (>68 years, HR: 1.59, P = 0.008), type of M protein (IgD, HR: 2.69, P = 0.001), R-ISS stage (3 *vs*. 1–2, HR: 1.53, P = 0.017), bone marrow plasma cell percentage (>30%, HR: 1.73, P = 0.001), spleen (enlarged, HR: 1.67, P = 0.004), and the state of EM (>1 site of EMB or EME, HR: 2.73, P = 0.002; >1 site of EMB with EME, HR: 12.48, P < 0.001) were independent prognostic factors of patients with PFS (**Table 3**). The patient’s age (>68 years, HR: 1.64, P = 0.022), Durie-Salmon stage (3B *vs*. 1–3A, HR: 1.93, P = 0.016), R-ISS stage (3 *vs*. 1-2, HR: 1.76, P = 0.017), bone marrow plasma cell percentage (>30%, HR: 1.68, P = 0.015), spleen (enlarged, HR: 1.91, P = 0.003), and the state of EM (>1 site of EMB or EME, HR: 3.16, P = 0.002; >1 site of EMB with EME, HR: 6.88, P < 0.001) were independent prognostic factor of OS (**Table 4**).

### Effect of Treatment on Survival of Patients With EMM

#### Induction Therapy Regimens

The cohort of 80 EMM patients was treated with bortezomib-based chemotherapy (**Table 1**). Survival analysis indicated that the median PFS of patients treated with PD, PAD, PCD, and PTD regimens were 9.6 (95% CI: 6.9–10.3) months, 24.3 (95% CI: 0–49.4) months, 18.8 (95% CI: 0–38.6) months, and 13.5 (95% CI: 11.5–15.6) months. PAD may have slightly better PFS, but the difference has not statistically significant (P = 0.104). On the other hand, the median OS of patients receiving PD, PAD, PCD, and PTD regimens was 18.8 (95% CI: 13.2–24.5) months, not reached (NR), 43.6 (95% CI: 29.3–58.0) months, and 28.5 (95% CI: 19.1–37.9), respectively; the difference was statistically significant (P = 0.013). Furthermore, PAD regimens may have better OS, but further analysis suggested that PD regimens have worse OS than PAD and PCD (P = 0.004, 0.011), but no difference was detected in PTD regimens (P = 0.586); also, no statistically significant difference was observed between PAD and PCD or PTD regimens (P = 0.153, 0.125).

#### ASCT

**Figure 3:**
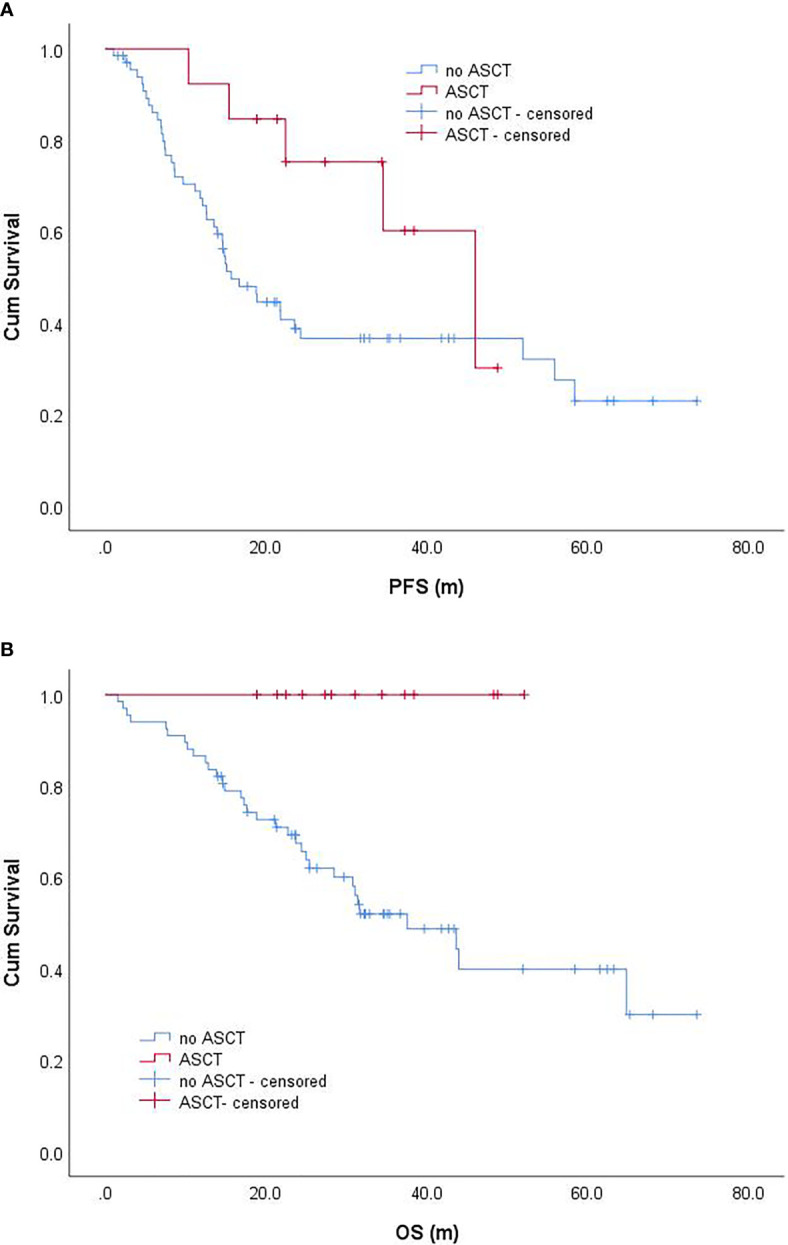
Progression-free survival **(A)** and overall survival **(B)** of EMM patients with and without ASCT. progression-free survival; OS, overall survival.

Among 80 EMM patients, 13 patients received ASCT. The median PFS of patients receiving ASCT was 46.0 (95% CI: 29.0–63.1) months, while the median PFS of patients not receiving ASCT was 15.7 (95% CI: 11.2–20.2) months; patients receiving ASCT had better PFS, but not statistically significant (P = 0.073) (**Figure 3A**). The median OS of patients receiving ASCT was NR, while the median OS of patients not receiving ASCT was 37.6 (95% CI: 23.0–52.1) months. The median OS of patients who received ASCT was significantly better than that of those who did not receive ASCT (P = 0.006) (**Figure 3B**). Further analysis of EMM patients aged <68 years revealed that the median PFS of patients receiving ASCT and those not receiving ASCT were 46.0 (95% CI: 29.0–63.1) months and 18.9 (95% CI: 11.9–25.9) months, respectively, and the former might have a better PFS, but not statistically significant (P = 0.132). The median OS of patients receiving ASCT and those not receiving ASCT were NR and 43.6 months (95% CI: 21.2–66.1), respectively, and the patients receiving ASCT had significantly better OS than those not receiving ASCT (P = 0.014).

## Discussion

Based on the definition and evaluation methods, the reported incidence of EMM was also inconsistent. A large number of studies are based on general imaging, such as MR, CT, X-ray, B-ultrasound, and routine physical examination. A study of 2,332 patients from eight clinical trials, enrolled from 2005 to 2015, indicated that the incidence of EM in patients with newly diagnosed MM was 11.4%; the incidence of EMB was 243 (10.4%), while the incidence of EME was only 12 (0.5%) (4). Among them, 195 (73%) cases were single involvement, and 60 (22%) cases showed multiple involvement. In another study (2005–2014), the data of 3,744 patients who received ASCT suggested that the total incidence of EMM at the initial diagnosis was 782 (18.2%) cases, of which 543 (14.5%) were EMB and 139 (3.7%) were EME; the incidence of EMM increased from 6.5% in 2005 to 23.7% in 2014 (5). The reason may be increased sensitivity of image detection and that a large number of patients have adopted PET/CT in the later stage. Among our 357 patients with newly diagnosed MM, 80 cases were EMM, with an incidence rate of 22.4%. If PET/CT was used, the detection rate was 25.9%, while for patients who did not use PET/CT, the detection rate was only 14.2% (P = 0.015). PET/CT imaging might detect additional cases of EMM; however, several studies reported that PET/CT could not increase the detection rate of EMM (12–14). The Recent International Myeloma Working Group guidelines recommended PET/CT for the newly diagnosed and relapsed/refractory MM to determine the extent of bone damage and extramedullary involvement (15).

Furthermore, some patients did not have splenic space-occupying lesions, but the reason for spleen enlargement is not yet clarified. In addition to the involvement of MM cells, this phenomenon could also be due to splenic blood stasis (such as water sodium retention and right heart dysfunction), amyloidosis (16), other hereditary metabolic cells (Gaucher disease) (17), and many other unexplained causes of enlarged spleen. In the current study, a total of 84 patients had spleen enlargement; however, there was no significant difference in the detection rate of splenomegaly between the EMM group (26.3%) and the non-EM group (22.7%), rendering it difficult to confirm whether splenomegaly belongs to plasma cell involvement. The present study also confirmed that splenomegaly is related to poor PFS and OS. However, due to the lack of research on this aspect, the reason is unclear, thereby necessitating in-depth investigation.

EMM is a poor prognostic factor for MM, usually with a high tumor load, severe anemia, thrombocytopenia, hypercalcemia, and renal insufficiency, accompanied by increased serum LDH and β2-microglobulin levels (2). The current data showed that several EMM patients had higher serum LDH than normal, and the Hgb level of EMM patients was significantly higher than that of patients without EM (P = 0.002). In addition, the proportion of bone marrow plasma cells in EMM patients was lower than that in patients without EM, albeit not significantly (P = 0.09). Simultaneously, compared with MM patients, more EMM patients were in R-ISS stage I (20.5 *vs*. 7.9%, P = 0.009). Montefusco et al. (4) analyzed the clinical characteristics of 267 EMM patients and 2,065 patients without EM; patients with EMM had higher Hgb levels and a lower percentage of bone marrow plasma cells than those without EM; similar results have been reported previously (18, 19). These phenomena suggested that EMM is a specific clinical feature, and the final influence on the outcome of these patients could be attributed to EM rather than tumor burden.

Most of the patients with EMM have a higher incidence of genetic abnormalities related to poor prognosis, such as t (4;14) and t (14;16), which increase from 13 and 3% to 23 and 8%, respectively, and 1q21 amplification increase from 34 to 54% (20, 21). However, according to Deng et al., the above abnormalities did not increase in newly diagnosed EMM patients compared to MM, while del 17p involving the *TP53* gene showed a significant increase in EMM (34.5 *vs* 11.9%) (22). The current data did not find that the incidence of 17p deletion, 1q amplification, and chromosome 14 translocation increased in EMM. Furthermore, EMM has different characteristics from MM at the molecular level, which is mainly involved in the downregulation of cell adhesion and chemokines, while the upregulation of cell migration, proliferation, and immune escape (2, 23, 24). In addition, the gene expression profiles showed that EMM had high-risk characteristics, such as the proportion of MF subtype and PR subtype, which were significantly higher than those of patients without EM (12, 25, 26).

Due to the lack of prospective research data, there is no standard recommended treatment for EMM patients at present; however, it is speculated that a combination of drugs with different mechanisms of action [such as lenalidomide-bortezomib-dexamethasone (RVD)] and ASCT might be an optimal approach (2, 3). Patients with EMM should avoid the use of suboptimal induction therapy while focusing on adequate supportive care, especially in elderly patients. Due to economic reasons, the patients in the current study mainly received bortezomib-based treatment regimens instead of combining bortezomib and lenalidomide. Nonetheless, the prognosis of EMM patients was poor (4–6, 12), especially EME, which differed from that of EMB with respect to biological characteristics, and exhibited worse PFS and OS (4–6, 14). Beksac et al. (6) summarized the data of 226 EMM patients and found that newly diagnosed EME patients had worse OS (NR *vs*. 46.5 m) than EMB patients, and ASCT could at least partially improve the patients’ OS. On the other hand, Montefusco et al. reported that newly diagnosed EMM patients and patients without EM treated with bortezomib and/or lenalidomide had similar PFS, with a median PFS of 25.3 months *vs*. 25.2 months (P = 0.46). Therefore, the treatment with new drugs might overcome the impact of EMM on PFS in newly diagnosed patients but could still affect the OS, thereby deeming it as an independent poor prognostic factor of OS (4). Our study also suggested that EMM has worse PFS and OS than patients without EM. Even if bortezomib-based treatment was administered, the lack of combination therapy with lenalidomide and the low proportion of patients receiving ASCT could be ascribed to the failure to overcome the adverse effects on PFS.

Intriguingly, the current study suggested that most patients with EMB had only one involved site (53/80), while all EME patients were accompanied by EMB involvement, and most (9/10) were accompanied by multiple sites of EMB involvement. Gagelmann et al. (5) also suggested that EME has multiple sites of involvement. Patients with EME and patients with multiple sites of involvement have worse survival, while those with single-site EMB have survival similar to patients without EM, but the single site of EME was associated with poor outcome (5). We divided EMM into a single site of EMB, multiple sites of EMB or EME (only one EME in this group), and multiple sites of EMB with EME and found that single site of EMB and patients without EM had similar PFS and OS, while multiple sites of EMB and/or EME were independent prognostic factors for MM patients.

Even in this era of new drugs, ASCT has an irreplaceable role in MM (1). Although ASCT is used in EMM patients, it has a worse survival than patients without EM, but both EMB and EME patients can benefit from ASCT. In a previous study with 51.5% of primary EMM (6), the PFS of patients receiving ASCT was 49 months (EMB: 51.7 months, EME: 46.5 months, P = NS), and the median PFS of patients not receiving ASCT was only 28.1 months (P < 0.001); the median OS was 79.5 months in the ASCT group and 34.7 months in the non-ASCT group. Therefore, ASCT was deemed an independent prognostic factor for the survival of patients with EMM. The current study demonstrated that patients who received ASCT had better OS (NS *vs*. 37.6 months, P = 0.006). In addition, the data suggested that patients who received dual ASCT had a lower risk of death than patients who received single ASCT (HR: 1.46, P = 0.02) (27).

In this study, PET/CT was used to screen the whole body EMM of MM during diagnosis. However, in the absence of pathological confirmation in some organs, such as the spleen, especially in the absence of space-occupying lesions, could not be confirmed as extramedullary involvement of MM, which might have some impact on the reliability of the data. In addition, these data were obtained from the retrospective analysis, and <20% of patients received ASCT. Therefore, we propose a prospective clinical study that would use PET/CT or multiple sites of MR combined with pathological biopsy diagnostic methods and the currently recommended multi-drug combination therapy regimen plus ASCT to provide evidence-based data for the diagnosis and treatment of EMM.

## Data Availability Statement

The original contributions presented in the study are included in the article/supplementary material. Further inquiries can be directed to the corresponding author.

## Ethics Statement

The studies involving human participants were reviewed and approved by the Ethics Committee of the First Affiliated Hospital of Zhejiang University. The patients/participants provided their written informed consent to participate in this study. Written informed consent was obtained from the individual(s) for the publication of any potentially identifiable images or data included in this article.

## Author Contributions

JH wrote a part of the manuscript, reviewed literature, managed the patients, and collected clinical data. XY translated the manuscript, reviewed, and wrote a part of the manuscript. DH, YZ, GZ, and EZ reviewed and wrote a part of the manuscript and provided suggestions. XH, YY, LY, JC and WW managed the patients, reviewed, and wrote a part of the manuscript. ZC wrote a part of the manuscript, reviewed the literature, and provided suggestions. All authors reviewed the manuscript. All authors contributed to the article and approved the submitted version.

## Funding

This study was supported by the Youth Program of National Natural Science Foundation of China (grant numbers 81900209, 81800202) and the Natural Science Foundation of Zhejiang Province (grant number LQ21H160015).

## Conflict of Interest

The authors declare that the research was conducted in the absence of any commercial or financial relationships that could be construed as a potential conflict of interest.

## References

[1] Kumar SK Rajkumar V Kyle RA van Duin M Sonneveld P Mateos MV. Multiple Myeloma. Nat Rev Dis Primers (2017) 3:17046. 10.1038/nrdp.2017.46 28726797

[2] Bhutani M Foureau DM Atrash S Voorhees PM Usmani SZ. Extramedullary Multiple Myeloma. Leukemia (2020) 34(1):1–20. 10.1038/s41375-019-0660-0 31776467

[3] Rosinol L Beksac M Zamagni E Van de Donk N Anderson KC Badros A. Expert Review on Soft-Tissue Plasmacytomas in Multiple Myeloma: Definition, Disease Assessment and Treatment Considerations. Br J Haematol (2021). 10.1111/bjh.17338 33724461

[4] Montefusco V Gay F Spada S De Paoli L Di Raimondo F Ribolla R. Outcome of Paraosseous Extra-Medullary Disease in Newly Diagnosed Multiple Myeloma Patients Treated With New Drugs. Haematologica (2020) 105(1):193–200. 10.3324/haematol.2019.219139 31221778 PMC6939525

[5] Gagelmann N Eikema DJ Iacobelli S Koster L Nahi H Stoppa AM. Impact of Extramedullary Disease in Patients With Newly Diagnosed Multiple Myeloma Undergoing Autologous Stem Cell Transplantation: A Study From the Chronic Malignancies Working Party of the EBMT. Haematologica (2018) 103(5):890–7. 10.3324/haematol.2017.178434 PMC5927971 29419433

[6] Beksac M Seval GC Kanellias N Coriu D Rosinol L Ozet G. A Real World Multicenter Retrospective Study on Extramedullary Disease From Balkan Myeloma Study Group and Barcelona University: Analysis of Parameters That Improve Outcome. Haematologica (2020) 105(1):201–8. 10.3324/haematol.2019.219295 PMC6939516 31278209

[7] Touzeau C Moreau P. How I Treat Extramedullary Myeloma. Blood (2016) 127(8):971–6. 10.1182/blood-2015-07-635383 26679866

[8] He J He D Han X Zheng G Wei G Zhao Y. Bortezomib-Based Regimens for Newly Diagnosed Multiple Myeloma in China: A Report of 12-Year Real-World Dat. Front Pharmacol (2020) 11:561601. 10.3389/fphar.2020.561601 33362538 PMC7759685

[9] Durie BG Harousseau JL Miguel JS Blade J Barlogie B Anderson K. International Uniform Response Criteria for Multiple Myeloma. Leukemia (2006) 20(9):1467–73. 10.1038/sj.leu.2404284 16855634

[10] Kumar S Paiva B Anderson KC Durie B Landgren O Moreau P. International Myeloma Working Group Consensus Criteria for Response and Minimal Residual Disease Assessment in Multiple Myeloma. Lancet Oncol (2016) 17(8):E328–E46. 10.1016/S1470-2045(16)30206-6 27511158

[11] Rajkumar SV. Multiple Myeloma: Every Year a New Standard? Hematol Oncol (2019) 37(Suppl 1):62–5. 10.1002/hon.2586 PMC6570407 31187526

[12] Usmani SZ Heuck C Mitchell A Szymonifka J Nair B Hoering A. Extramedullary Disease Portends Poor Prognosis in Multiple Myeloma and Is Over-Represented in High-Risk Disease Even in the Era of Novel Agents. Haematologica (2012) 97(11):1761–7. 10.3324/haematol.2012.065698 PMC3487453 22689675

[13] Short KD Rajkumar SV Larson D Buadi F Hayman S Dispenzieri A. Incidence of Extramedullary Disease in Patients With Multiple Myeloma in the Era of Novel Therapy, and the Activity of Pomalidomide on Extramedullary Myeloma. Leukemia (2011) 25(6):906–8. 10.1038/leu.2011.29 PMC3736849 21350560

[14] Moreau P Attal M Caillot D Macro M Karlin L Garderet L. Prospective Evaluation of Magnetic Resonance Imaging and [(18)F]Fluorodeoxyglucose Positron Emission Tomography-Computed Tomography at Diagnosis and Before Maintenance Therapy in Symptomatic Patients With Multiple Myeloma Included in the IFM/DFCI 2009 Trial: Results of the IMAJEM Stud. J Clin Oncol (2017) 35(25):2911–8. 10.1200/JCO.2017.72.2975 PMC5578392 28686535

[15] Cavo M Terpos E Nanni C Moreau P Lentzsch S Zweegman S. Role of (18)F-FDG PET/CT in the Diagnosis and Management of Multiple Myeloma and Other Plasma Cell Disorders: A Consensus Statement by the International Myeloma Working Group. Lancet Oncol (2017) 18(4):e206–e17. 10.1016/S1470-2045(17)30189-4 28368259

[16] Buzalewski J Fisher M Rambaran R Lopez R. Splenic Rupture Secondary to Amyloid Light-Chain (AL) Amyloidosis Associated With Multiple Myeloma. J Surg Case Rep (2019) 2019(3):rjz021. 10.1093/jscr/rjz021 30949329 PMC6439512

[17] Chadha J Sahai T Schwimmer J Shani D. A 30-Year-Old Carrier of Gaucher Disease With Multiple Myeloma. Case Rep Oncol Med (2019) 2019:6469196. 10.1155/2019/6469196 30906609 PMC6393880

[18] Varettoni M Corso A Pica G Mangiacavalli S Pascutto C Lazzarino M. Incidence, Presenting Features and Outcome of Extramedullary Disease in Multiple Myeloma: A Longitudinal Study on 1003 Consecutive Patients. Ann Oncol (2010) 21(2):325–30. 10.1093/annonc/mdp329 19633044

[19] Wu P Davies FE Boyd K Thomas K Dines S Saso RM. The Impact of Extramedullary Disease at Presentation on the Outcome of Myeloma. Leuk Lymphoma (2009) 50(2):230–5. 10.1080/10428190802657751 19197724

[20] Avet-Loiseau H Malard F Campion L Magrangeas F Sebban C Lioure B. Translocation T(14;16) and Multiple Myeloma: Is it Really an Independent Prognostic Factor? Blood (2011) 117(6):2009–11. 10.1182/blood-2010-07-295105 20962323

[21] Chang H Qi X Yeung J Reece D Xu W Patterson B. Genetic Aberrations Including Chromosome 1 Abnormalities and Clinical Features of Plasma Cell Leukemia. Leuk Res (2009) 33(2):259–62. 10.1016/j.leukres.2008.06.027 18676019

[22] Deng S Xu Y An G Sui W Zou D Zhao Y. Features of Extramedullary Disease of Multiple Myeloma: High Frequency of P53 Deletion and Poor Survival: A Retrospective Single-Center Study of 834 Cases. Clin Lymphoma Myeloma Leuk (2015) 15(5):286–91. 10.1016/j.clml.2014.12.013 25640025

[23] Jelinek T Bezdekova R Zatopkova M Burgos L Simicek M Sevcikova T. Current Applications of Multiparameter Flow Cytometry in Plasma Cell Disorders. Blood Cancer J (2017) 7(10):e617. 10.1038/bcj.2017.90 29053157 PMC5678219

[24] Rihova L Vsianska P Bezdekova R Zdenek A Miroslav P Tomas J. Identification of Phenotype Profile Related to the Extramedullary Involvement in Multiple Myeloma Relapse. Blood (2016) 128(22):5653. 10.1182/blood.V128.22.5653.5653

[25] Billecke L Murga Penas EM May AM Engelhardt M Nagler A Leiba M. Cytogenetics of Extramedullary Manifestations in Multiple Myeloma. Br J Haematol (2013) 161(1):87–94. 10.1111/bjh.12223 23368088

[26] Zhou Y Barlogie B Shaughnessy JD Jr. The Molecular Characterization and Clinical Management of Multiple Myeloma in the Post-Genome Era. Leukemia (2009) 23(11):1941–56. 10.1038/leu.2009.160 PMC3686133 19657360

[27] Gagelmann N Eikema DJ Koster L Caillot D Pioltelli P Lleonart JB. Tandem Autologous Stem Cell Transplantation Improves Outcomes in Newly Diagnosed Multiple Myeloma With Extramedullary Disease and High-Risk Cytogenetics: A Study From the Chronic Malignancies Working Party of the European Society for Blood and Marrow Transplantation. Biol Blood Marrow Transplant (2019) 25(11):2134–42. 10.1016/j.bbmt.2019.07.004 31288095

